# Nanoencapsulation of MDM2 Inhibitor RG7388 and Class-I HDAC Inhibitor Entinostat Enhances their Therapeutic Potential Through Synergistic Antitumor Effects and Reduction of Systemic Toxicity

**DOI:** 10.1021/acs.molpharmaceut.3c00926

**Published:** 2024-02-09

**Authors:** Anas Abed, Michelle K. Greene, Alhareth A. Alsa’d, Andrea Lees, Andrew Hindley, Daniel B Longley, Simon S McDade, Christopher J. Scott

**Affiliations:** † The Patrick G Johnston Centre for Cancer Research, School of Medicine, Dentistry and Biomedical Sciences, Queen’s University Belfast, 97 Lisburn Road, Belfast BT9 7AE, United Kingdom; ‡ Pharmacological and Diagnostic Research Centre, Faculty of Pharmacy, Al-Ahliyya Amman University, Amman 19111, Jordan; § School of Pharmacy, Queen’s University Belfast, 97 Lisburn Road, Belfast BT9 7BL, United Kingdom; ∥ Clinical Haematology, Belfast City Hospital, 97 Lisburn Road, Belfast, BT9 7AB, United Kingdom

**Keywords:** cancer, nanoparticles, Entinostat, nutlin, toxicity, combination therapy

## Abstract

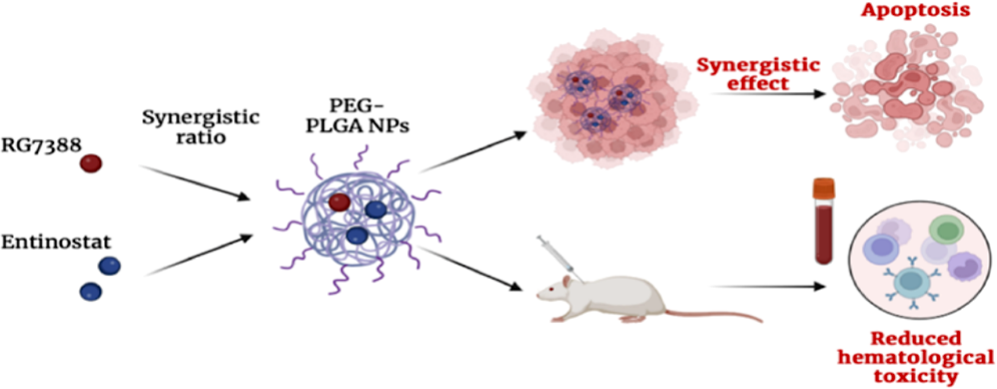

Inhibitors of the p53–MDM2 interaction such as RG7388 have been developed to exploit latent tumor suppressive properties in p53 in 50% of tumors in which p53 is wild-type. However, these agents for the most part activate cell cycle arrest rather than death, and high doses in patients elicit on-target dose-limiting neutropenia. Recent work from our group indicates that combination of p53–MDM2 inhibitors with the class-I HDAC inhibitor Entinostat (which itself has dose-limiting toxicity issues) has the potential to significantly augment cell death in p53 wild-type colorectal cancer cells. We investigated whether coencapsulation of RG7388 and Entinostat within polymeric nanoparticles (NPs) could overcome efficacy and toxicity limitations of this drug combination. Combinations of RG7388 and Entinostat across a range of different molar ratios resulted in synergistic increases in cell death when delivered in both free drug and nanoencapsulated formats in all colorectal cell lines tested. Importantly, we also explored the *in vivo* impact of the drug combination on murine blood leukocytes, showing that the leukopenia induced by the free drugs could be significantly mitigated by nanoencapsulation. Taken together, this study demonstrates that formulating these agents within a single nanoparticle delivery platform may provide clinical utility beyond use as nonencapsulated agents.

## Introduction

The tumor suppressive transcription factor p53 is a central regulator of the cellular stress response. Under normal homeostatic conditions, p53 protein is maintained at low steady-state levels by active degradative suppression by its negative regulator E3-Ligase MDM2.^1^ However, in response to stresses, such as DNA damage, this regulatory pathway is disrupted in favor of p53 stabilization and hyperactivation. In turn, this leads to knock-on effects on multiple target genes that regulate various processes, such as DNA damage repair, cell cycle arrest, and apoptosis (together with a range of other activities), underlying the p53’s tumor suppressive function.^2^

Due to the importance of MDM2 in suppressing p53 activity, much interest has focused on the development of small molecule agents that can disrupt the interaction of these two proteins. The best-characterized class of MDM2 antagonists is *cis*-imidazoline compounds termed ‘nutlins’ (Nutley Inhibitors) such as nutlin-3a, which is highly effective in stabilizing p53, but at least *in vitro* results in predominantly cell cycle arrest.^3^ Despite potency and specificity in preclinical studies, nutlin-3a was not progressed to clinical trials due to its poor pharmacokinetic properties and associated dose limiting toxicities (DLT), such as leukopenia and thrombocytopenia.^4^ Rather, second-generation MDM2 antagonists, such as the pyrrolidine RG7388, with improved pharmacokinetic properties and superior efficacy were developed.^5,6^ Despite these improvements, (likely on-target) toxicity still remains a problem with second-generation MDM2 antagonists,^7^ with RG7388 found to cause myelosuppression and subsequent leukopenia and thrombocytopenia in acute myeloid leukemia (AML) patients.^8,17^

A further constraint of MDM2 antagonists is their limited efficacy against solid tumors. While they induce cell cycle arrest, tumor cells often exhibit resistance to apoptosis.^157^ Recently, we reported that, in fact, p53 activation directly induces the antiapoptotic pseudocaspase FLIP, which plays an important role in suppressing apoptosis in response to MDM2 antagonists in colorectal cancer (CRC) cells.^9^ Importantly, for this study, we also found that the class I histone deacetylase (HDAC) inhibitor Entinostat could enhance the apoptotic effects of nutlin-3a, largely through FLIP downregulation.^10^ Thus, the combination of Entinostat and a MDM2 antagonist could have clinical utility in p53-wild-type CRC. However, given the dose-limiting toxicities of RG7388 and Entinostat (neutropenia, thrombocytopenia, leukopenia, gastrointestinal, cardio, and metabolic effects),^11−13^ such a combination may benefit from new approaches to codeliver these agents to tumors at concentrations at which they can synergize to induce cancer cell death.

In recent decades, the application of nanotechnology has attracted much attention as a drug delivery approach for anticancer agents. This approach has the potential to overcome dose-limiting toxicities associated with anticancer agents and can also allow for simultaneous delivery of coentrapped payloads at synergistic levels to tumors.^14,15^ In keeping with these potential benefits, this study aimed to investigate whether dual loading of both RG7388 and Entinostat within PEGylated PLGA nanoparticles could be achieved at optimal ratios, leading to synergistic induction of cell death in CRC cultures. Moreover, we aimed to demonstrate that the nanoencapsulation of RG7388 and Entinostat could largely protect against hematological toxicity induced by these agents.

## Materials and Methods

### Cell Culture

HCT116 p53^+/+^ and p53^–/–^ colorectal cancer cell lines were a kind gift from Prof Bert Vogelstein’s laboratory (John Hopkins Centre, Baltimore^16^). LoVo and RKO colorectal cancer cell lines were obtained from the American Type Culture Collection (ATCC). All cells were cultured in high glucose Dulbecco’s Modified Eagle Medium (DMEM) (Gibco) supplemented with 10% fetal bovine serum (FBS) (Gibco), 1% Penicillin/Streptomycin (Pen/Strep) (5000 units/mL penicillin and 5000 units/mL streptomycin) (Gibco), 2 mM l-glutamine (Gibco), and 1 mM sodium pyruvate (Gibco).

### Assessment of Cell Viability and Apoptosis

Cell viability was assessed via CellTiter-Glo (CTG) assay (Promega) according to the manufacturer’s instructions, and the viability was expressed relative to that of untreated control cells. Apoptotic cell death was examined via FITC-Annexin V and propidium iodide (PI) staining. Briefly, 4 × 10^5^ HCT116 p53^+/+^, RKO, and LoVo cells were seeded in 6-well plates and subjected to drug treatments for 72 h. At the designated end point, the cells were transferred to 15 mL tubes. The cells were pelleted by centrifugation at 500*g* for 5 min and then incubated with 300 μL of 1× binding buffer (BD biosciences), containing 3 μL FITC-Annexin V and 2 μL of 50 μg/mL propidium iodide (PI) for 15 min prior to analysis on a BD Accuri C6 plus flow cytometer. Gates were set to exclude debris initially and then to discriminate between Annexin V/PI negative (live cells), PI-only positive (necrotic), Annexin V-only positive (early apoptotic), and Annexin V/PI positive (late apoptotic) cells.

### Nanoparticle Formulation

NPs were prepared in 20 mg batches by the nanoprecipitation method using a blend of 15 mg of PLGA 502H (Sigma) and 5 mg of PEG–PLGA copolymer (5000:10,000 mPEG:PLGA (Akina Inc.)). The polymer was dissolved in 1 mL of acetone (organic phase) and injected into an aqueous phase containing 0.01% Pluronic F-68 nonionic surfactant (ThermoFisher Scientific) in a dropwise manner while stirring. To prepare drug-loaded NPs, 1 mg of RG7388, Entinostat, or their combination was dissolved in 100 μL of DMSO prior to addition into the organic phase. The resultant suspension was stirred overnight to allow acetone evaporation. NPs were then purified by three wash-spin cycles at 16,000*g* for 15 min and resuspended in phosphate buffered saline (PBS) for characterization and cell work. To study nanoparticle uptake, the polymer blend was modified by adding 1 mg of PLGA-Rhodamine B (lactide:glycolide 50:50) (Sigma-Aldrich) to 15 mg of PLGA 502H and 5 mg of PEG–PLGA copolymer, and NPs were prepared using the same method.

### Nanoparticle Characterization

NPs were assessed in terms of size and polydispersity index (PDI) using a NanoBrook Omni instrument (Brookhaven Instruments corporation). The NPs were resuspended at a concentration of 0.2 mg/mL in PBS and transferred to a cuvette prior to analysis. The morphology and size distribution of the NPs were assessed using scanning electron microscopy (SEM). The NPs were washed and resuspended in deionized water (dH_2_O) at a concentration of 5 mg/mL. Next, 10 μL of NPs was added onto double-sided copper tape fixed to an aluminum stub, sputter coated with gold, and then imaged using a FEI Quanta 250 FEG-Environmental Scanning Electron Microscope (E-SEM).

### Assessing the Entinostat and RG7388 Drug Entrapment

The amount of RG7388 and Entinostat within NPs was assessed using high performance liquid chromatography (HPLC) and absorbance spectrometry, respectively. The NP pellet was lysed using a mixture of 1:1 acetonitrile and dimethyl sulfoxide (DMSO) to release any entrapped drug. RG7388 entrapment was detected by HPLC using a C18 reverse phase column (Phenomenex, 150 × 4.6 mm, 5 μM). The flow rate was set to be constant at 1 mL/min at 25 °C. 30 μL of 1 mg/mL of sample was injected per run, and the absorbance was detected at 273 nm and compared to a series of standards prepared by spiking known amounts of free RG7388 into blank NPs (BNPs) in 1:1 acetonitrile:DMSO. Entinostat entrapment was quantified by measurement of absorbance at 330 nm using a plate reader (Biotek) and again compared to a series of standards prepared by spiking known amounts of free Entinostat into BNPs in 1:1 acetonitrile:DMSO (Supplementary Figure 1). Drug loading was calculated using the formula below.
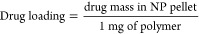


### Nanoparticle Uptake Study

Nanoparticle uptake was assessed using flow cytometry analysis in addition to confocal microscopy. HCT116 cells were seeded at a density of 4 × 10^5^ per well in a 6-well plate and incubated with 200 μg/mL of Rhodamine B-loaded NPs for 6 h. The media were then removed, and cells were washed three times with 2 mL of PBS. For flow cytometry analysis, gates were set to remove debris, and fluorescence was analyzed on 10,000 cells per sample using a BD Accuri C6 plus flow cytometer. For confocal microscopy analysis, HCT116 cells were washed with 2 mL of acid strip buffer (0.877 g of NaCl and 0.375 g of glycine in 100 mL of dH_2_O, pH 3) for 5 min, fixed with 2 mL of 4% w/v paraformaldehyde in PBS, and permeabilized with 2 mL of 0.5% v/v Triton X-100 in PBS. Next, cell nuclei were stained with DAPI (Vector Laboratories). Cells were imaged using a Leica SP-8 confocal microscope (Leica, UK) with a 1024 × 1024 frame. Images were analyzed using Leica LAS X software (Leica, UK).

### Drug Release Studies

Drug release from NPs was assessed using Slide-A-Lyzer dialysis cassettes 7 kDa (ThermoFisher Scientific). 20 mg of NPs was resuspended in 1 mL of PBS and injected into the dialysis cassette, which was immersed in a reservoir of 500 mL PBS containing 10% v/v FBS and 1% v/v Tween-20 at 37 °C under magnetic stirring. At specific time-points, NPs were collected and lysed in 1:1 acetonitrile:DMSO. Drug loading was then quantified as previously described. To calculate the cumulative release of the drug, the amount of drug still present within the NPs at each time point was subtracted from the initial total amount of drug loaded into the nanoparticles.

### *In Vivo* Toxicity Study

C57BL/6 mice (8–12 weeks old) were treated with dual-loaded NPs via intravenous injection (2 mg of polymer per animal in PBS), equivalent doses of free RG7388 and Entinostat mixed with BNPs via intraperitoneal injection (2 mg of polymer, Entinostat, and RG7388 per animal in 2% DMSO, 10% kolliphor, 30% PEG 400, and 58% saline), or corresponding vehicle controls on day 0 of the study. This dosing was repeated on day 5 of the study. Prior to commencing treatment, and also at 48 h after each dose, blood samples were collected via tail vein puncture using EDTA-coated capillary tubes (Greiner Bio-One). A complete blood count analysis (CBC) with white blood cells differential was then carried out in Belfast City Hospital using a XE-2100 automated hematology analyzer (Sysmex Corporation, Kobe, Japan) equipped with Sysmex Work Area Manager software. The body weight was monitored routinely to guarantee the animal welfare.

### Data Analysis

Statistical analyses were performed using Prism 8.0 software (Graphpad). Experimental results were compared using Student’s *t* test, one-way ANOVA, or two-way ANOVA where appropriate. Levels of significance were annotated as follows: * = *p* ≤ 0.05, ** = *p* ≤ 0.01, and *** = *p* ≤ 0.001. Combination indices (CI) were calculated according to the Chou and Talalay method using CompuSyn software. CI values of <1, =1, or >1 indicate synergism, additivity, or antagonism, respectively.

## Results

### Development and Characterization of RG7388-Loaded NPs

Given the documented pharmacokinetic and toxicity limitations of RG7388, our initial goal was to formulate the agent within a suitable biocompatible polymeric nanoparticle system. A nanoprecipitation method was adopted, leading to the generation of RG7388-loaded NPs (RGNPs) with an average diameter of 208.56 ± 6.13 nm, a monodisperse size distribution as indicated by a low PDI of 0.12 ± 0.04, and a negative zeta potential of −7.42 ± 2.31 (Table 1). Of note, the diameter of RGNPs was higher than that of blank nanoparticles (BNPs) likely due to the entrapment of RG7388 within the PLGA NPs. Quantification of drug loading (DL) by HPLC measurement revealed that 17.7 ± 1.2 μg of RG7388 was entrapped per mg of polymer. Release of RG7388 from the NPs followed a biphasic profile with an initial burst release of almost 55% of the entrapped RG7388 within the first 24 h followed by a slower drug release phase over the next 96 h (Supplementary Figure 2A). In contrast, free RG7388 was totally released after 6 h as expected.

**Table 1:** Drug Loading (Per mg of Polymer), Size, Polydispersity Index (PDI), and Zeta Potential of RG7388- and/or Entinostat-Loaded NPs^a^

formulation	RG7388 (μg/mg)	Entinostat (μg/mg)	size (nm)	PDI	zeta potential (mV)	RG7388: entinostat molar ratio
BNPs	-	-	169 ± 6.77	0.14 ± 0.06	–8.48 ± 1.31	-
RGNPs	17.67 ± 1.2	-	208.56 ± 6.13	0.12 ± 0.04	–7.42 ± 2.31	-
EnNPs	-	17.06 ± 0.88	209.43 ± 4.2	0.108 ± 0.05	–7.45 ± 3.65	-
DLNPs	13.87 ± 3.73	17.78 ± 2.93	226.2 ± 5.6	0.164 ± 0.04	–9.4 ± 2.3	1:2.1

a Data are expressed as mean ± standard deviation (SD) of three independent experiments.

To ensure that the RGNPs were capable of delivering their payload intracellularly, a NP uptake study was conducted using a fluorescent Rhodamine B-conjugated PLGA polymer, which was blended into the formulation to generate a NP that was trackable by flow cytometry and microscopy in a range of exemplar p53 wild-type CRC cell lines (RKO, LoVo, and HCT116 p53^+/+^). After 6 h coincubation, flow cytometry and confocal microscopy analyses revealed that the NPs were readily internalized as indicated by an increase in rhodamine fluorescence intensity with treated cells compared to untreated controls (Supplementary Figure 3).

**Figure 1:**
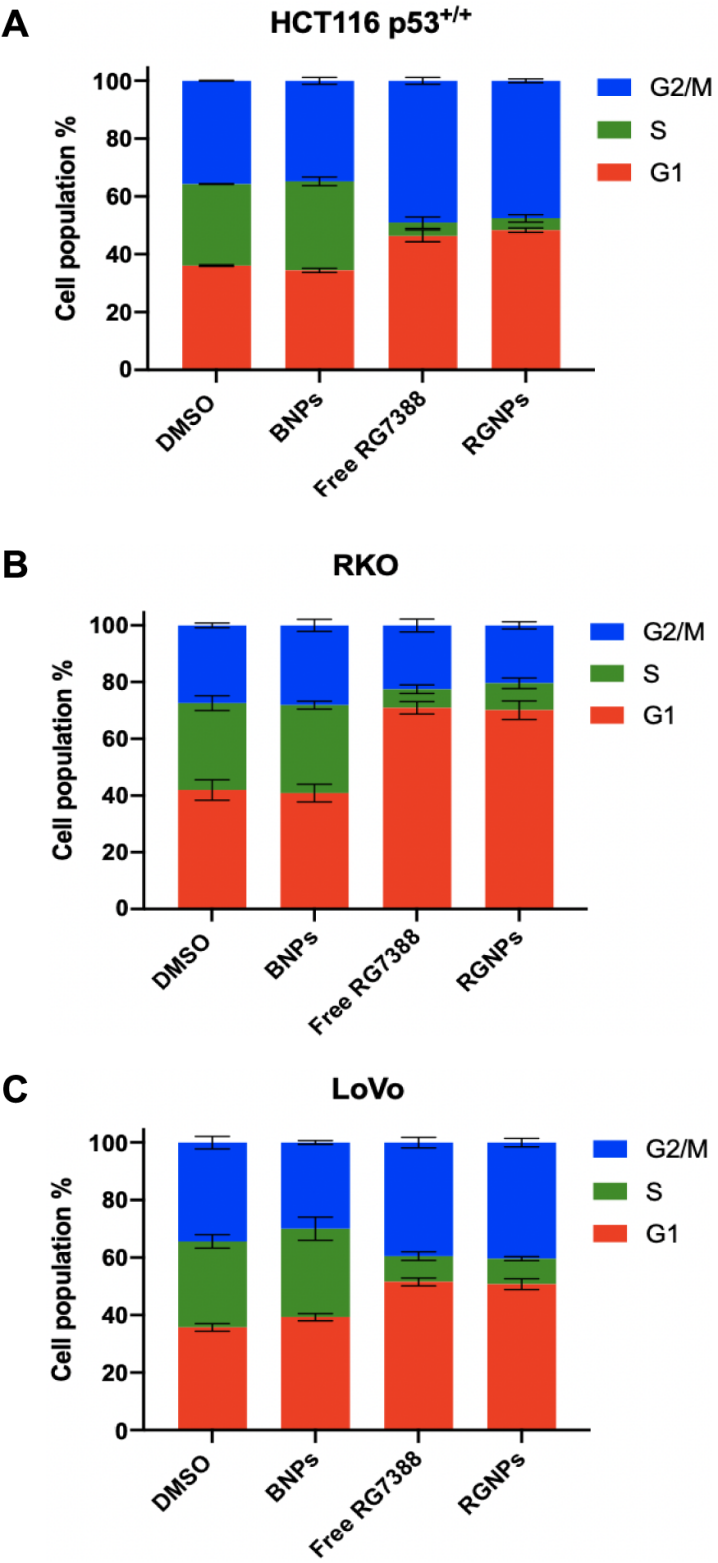
RGNPs induce cell cycle arrest at the G1 and G2/M phases in colorectal cancer cells. HCT116 p53^+/+^ (A), RKO (B), and LoVo (C) cell lines were treated with 1 μM of free RG7388, RGNPs (equating to 1 μM of RG7388), or BNPs (equating to polymer concentration of RGNPs) for 24 h prior to cell cycle analysis by flow cytometry. Data expressed as mean ± SD of three independent experiments.

Next to assess the pharmacological effect of RGNPs, cell cycle analyses were performed on the HCT116 p53^+/+^, LoVo, and RKO cell lines. The RGNPs attenuated cell cycle progression in these cell lines comparably to free drug treatment, as indicated by cell cycle arrest at the G1 and G2/M phases (Figure 1, Supplementary Figure 4). Moreover, these findings were further supported by CellTiter-Glo analyses, where all cell lines showed a similar level of sensitivity to RG7388 in both free and nanoencapsulated formats, confirming that the formulation process had no adverse effects on the potency of the RG7388 molecule (Supplementary Figure 5).

### Combination of RG7388 with Entinostat Enhances Cell Death in a p53 Dependent Manner

**Figure 2:**
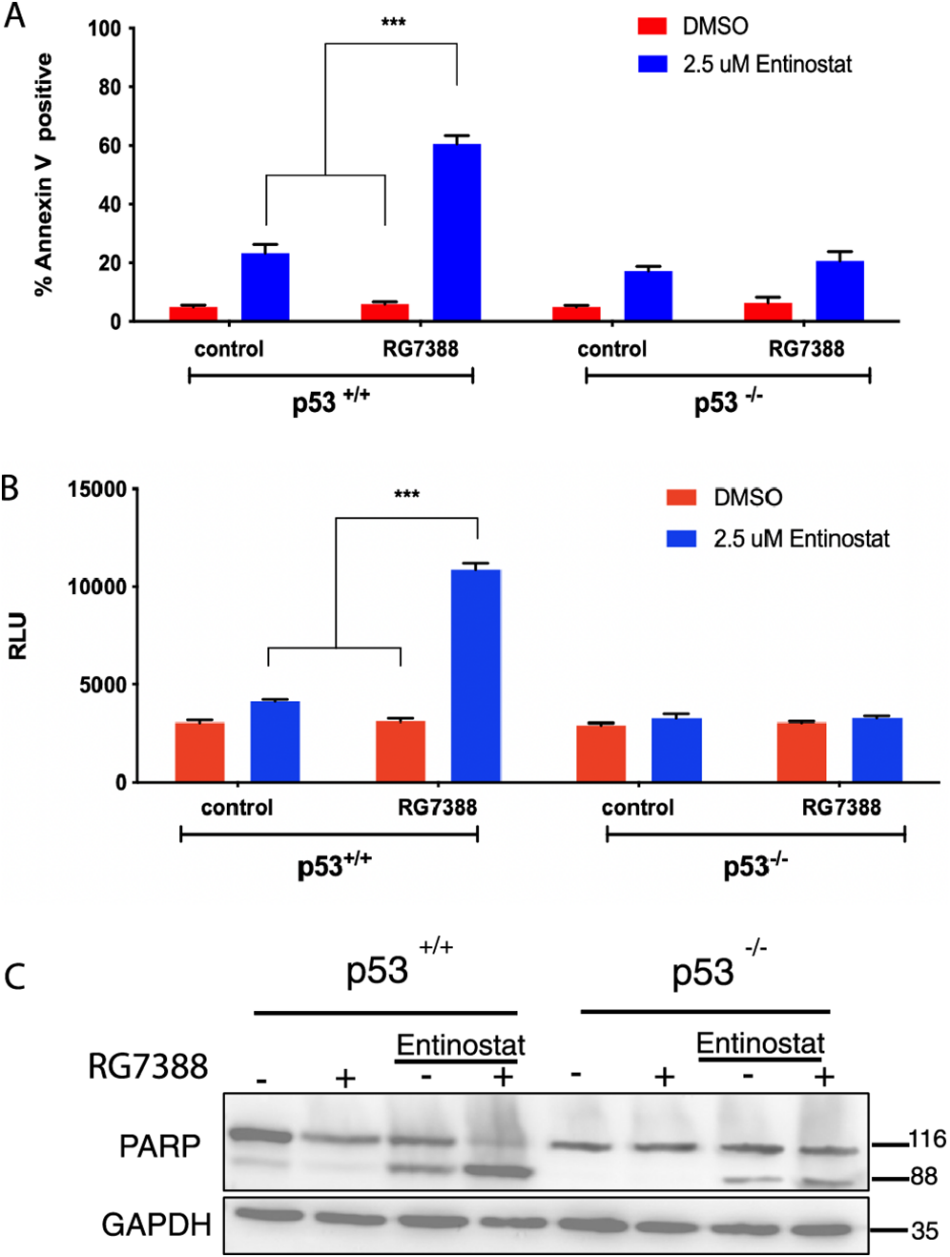
Entinostat synergizes with RG7388 to induce apoptosis in colorectal cancer cells. HCT116 p53 isogenic cells were treated with 1 μM RG7388, 2.5 μM Entinostat, or their combination, for 72 h prior to Annexin-V/PI flow cytometry analysis (A), assessment of caspase 3/7 activity (B), and Western blot analysis of protein expression (C). GAPDH was used as a loading control. Data expressed as mean ± SD of three independent experiments. ***p < 0.001 calculated by one-way ANOVA (Tukey posthoc).

Despite the successful development and application of RGNPs in inducing cell cycle arrest in cells bearing wild-type p53, we and others have demonstrated that MDM2 antagonists including RG7388 induce modest levels of apoptosis as single agents in colorectal models.^17^ Therefore, we next explored whether free RG7388-induced apoptosis could be augmented by combination with the class-I HDAC inhibitor Entinostat, which we have previously found to synergize with MDM2 antagonist nutlin-3A.^9^ To confirm this, HCT116 p53 wild-type (p53^+/+^) and isogenic null (p53^–/–^) cells were treated with either RG7388 or Entinostat alone, or in combination, prior to Annexin V/PI flow cytometry analysis of cell death (Figure 2A). As expected, at the concentrations analyzed in the HCT116 p53^+/+^ cells, RG7388 induced modest levels of apoptotic cell death. While single agent Entinostat resulted in higher levels of cell death than RG7388 as a single agent, the most significant effects were observed when the two drugs were combined, leading to significantly enhanced levels of apoptotic death (Figure 2A). In contrast, no significant increase in cell death with both agents in combination was observed in HCT116 p53^–/–^ cells, indicating the necessity for wild type p53 to achieve this effect. Apoptotic cell death correlated with a p53-dependent increase in caspase 3/7 activity (Figure 2B) accompanied by a significant increase in PARP cleavage following exposure (Figure 2C).

### Optimizing the Synergistic Ratio of RG7388 and Entinostat

**Figure 3:**
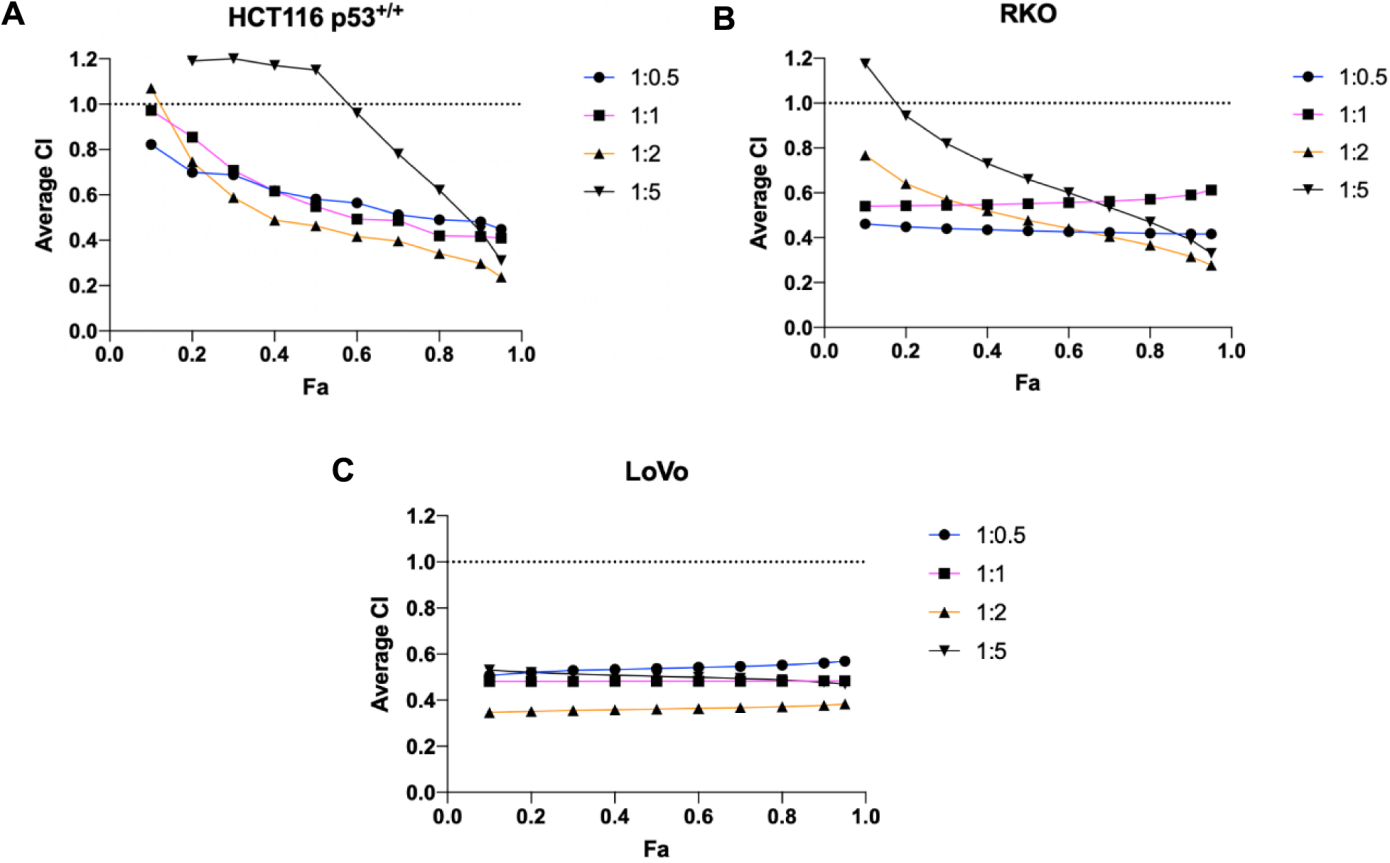
CI values for RG7388 and Entinostat cotreatment at different fractions affected (Fa) in colorectal cell lines. HCT116 p53^+/+^ (A), RKO (B), and LoVo (C) cell lines were treated with various molar ratios of RG7388:Entinostat (1:0.5, 1:1, 1:2, or 1:5). After 72 h, cell line sensitivity to treatment was assessed by CellTiter-Glo assay. Fa reflects the proportion of affected cells by the drug combination.

To determine whether the combined cytotoxic effects of RG7388 and Entinostat could be enhanced further through optimization of the drug treatment ratio, we next treated p53 wild-type lines (HCT116 p53^+/+^, RKO, and LoVo) with either single agents or with various molar ratios of RG7388:Entinostat (1:0.5, 1:1, 1:2, and 1:5) (Supplementary Figure 6). Measurement of the combination index (CI, Chou-Talalay method) indicated synergy (CI < 1) for all combinations of RG7388 and Entinostat except for 1:5 ratios (Figure 3). However, it was found that the 1:2 RG7388:Entinostat combination ratio resulted in the lowest CI at Fa = 0.95 compared to other combination ratios, suggesting that the 1:2 molar ratio induced the strongest synergistic effect (Table 2).

**Table 2:** CI Values for RG7388:Entinostat Cotreatment at Fa = 0.95

CI values for RG7388:Entinostat (72 h)				
RG7388:Entinostat molar ratio	1:0.5	1:1	1:2	1:5
HCT116 p53^+/+^	0.481	0.418	0.297	0.440
RKO	0.416	0.590	0.315	0.390
LoVo	0.561	0.483	0.378	0.478

### Development and Characterization of Entinostat-Loaded NPs

Following identification of the optimal ratio at which Entinostat synergistically augments the effects of RG7388 on viability, we explored coentrapping both drugs within a polymeric nanoparticle formulation. Initially, since Entinostat has poor aqueous solubility, we postulated that it could be entrapped using the same nanoprecipitation approach previously employed for RG7388. To assess this, nanoparticles entrapping Entinostat were developed by this process, and physicochemical characterization indicated that Entinostat-loaded NPs (EnNPs) had a size of 209.43 ± 4.2 nm, a low PDI of 0.108 ± 0.05, indicative of a uniform size distribution, and a negative zeta potential of −7.45 ± 3.65 (Table 1). Quantification of the amount of Entinostat within the polymeric NPs revealed that it was readily entrapped with a DL of 17.06 ± 0.88 μg of drug per milligram of polymer. Entinostat release from the NPs followed a biphasic pattern, with 60% initially released within the first 24 h followed by a sustained release phase over the next 96 h (Supplementary Figure 2B). The cytotoxicity of EnNPs was compared to that of free Entinostat in the colorectal cancer cell lines HCT116 p53^+/+^, LoVo, and RKO using Annexin-V/PI flow cytometry analysis. In all cell lines tested, EnNPs elicited a comparable cytotoxic effect to that observed with the free drug, confirming that the nanoformulation process did not affect its activity (Supplementary Figure 7).

### Preparation and Characterization of Dual-Loaded NPs

Following confirmation that Entinostat could be readily encapsulated via the same process as previously employed for RG7388, we next generated polymeric NPs coloaded with both agents at the optimal synergistic ratio (1:2). The dual drug-loaded nanoparticles (DLNPs) had an average diameter of 226.2 ± 5.6 nm, a low PDI of 0.164 ± 0.04 indicative of a uniform size distribution, and a negative zeta potential of −9.4 ± 2.3 (Table 1). SEM analysis additionally confirmed the monodispersity and the spherical morphology of the DLNPs (Supplementary Figure 8).

**Figure 4:**
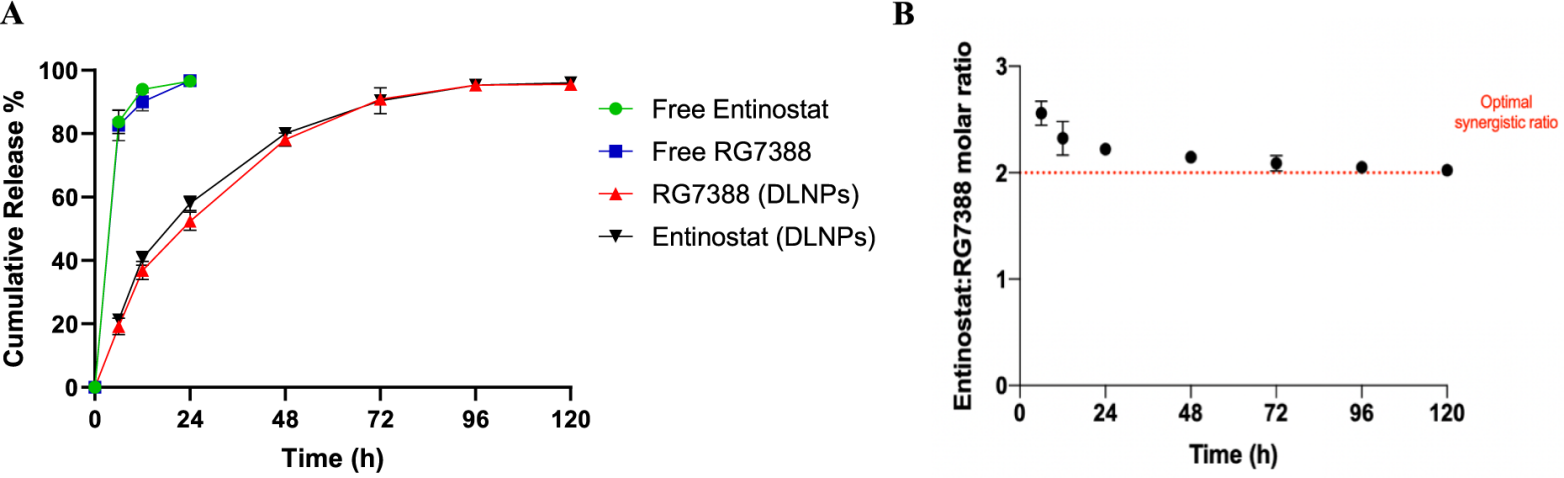
Assessment of drug release from DLNPs. (A) 20 mg of DLNPs was resuspended in 1 mL of PBS and then injected into a dialysis cassette, which was then immersed in PBS containing 10% FBS and 1% Tween-20 under constant stirring at 37 °C. At the indicated time points, the remaining NPs were removed, and RG7388 and Entinostat release was quantified using HPLC and UV–vis spectrophotometry, respectively. The release kinetics of free RG7388 and free Entinostat were also analyzed in parallel. (B) The data generated in (A) was used to calculate the molar ratio of drugs released from NPs at the indicated time points. Data represents mean ± SEM of three independent experiments.

Analysis of the cumulative release of RG7388 and Entinostat from the DLNPs was performed over a period of 120 h in PBS containing 10% FBS and 1% Tween-20 at 37 °C. Both agents showed a biphasic release pattern with an initial burst release of 53.6 ± 3.69% of RG7388 and 58 ± 2.87% of Entinostat during the first 24 h, followed by a slower release profile over the next 96 h (Figure 4 A). The RG7388: Entinostat molar ratio was also monitored throughout the study to ensure that both drugs were released at a synergistic ratio (Figure 4 B). This revealed that, on average, RG7388 and Entinostat were released at a ratio of 1:2.22 over the period of 120 h, which was almost equivalent to the optimal synergistic ratio empirically established. Importantly, these results showed that we were able to entrap RG7388 and Entinostat at an optimized molar ratio (1:2.1) and maintain drug release from the formulation at this ratio.

### Cell Death Assessment Following Treatment with the DLNPs

**Figure 5:**
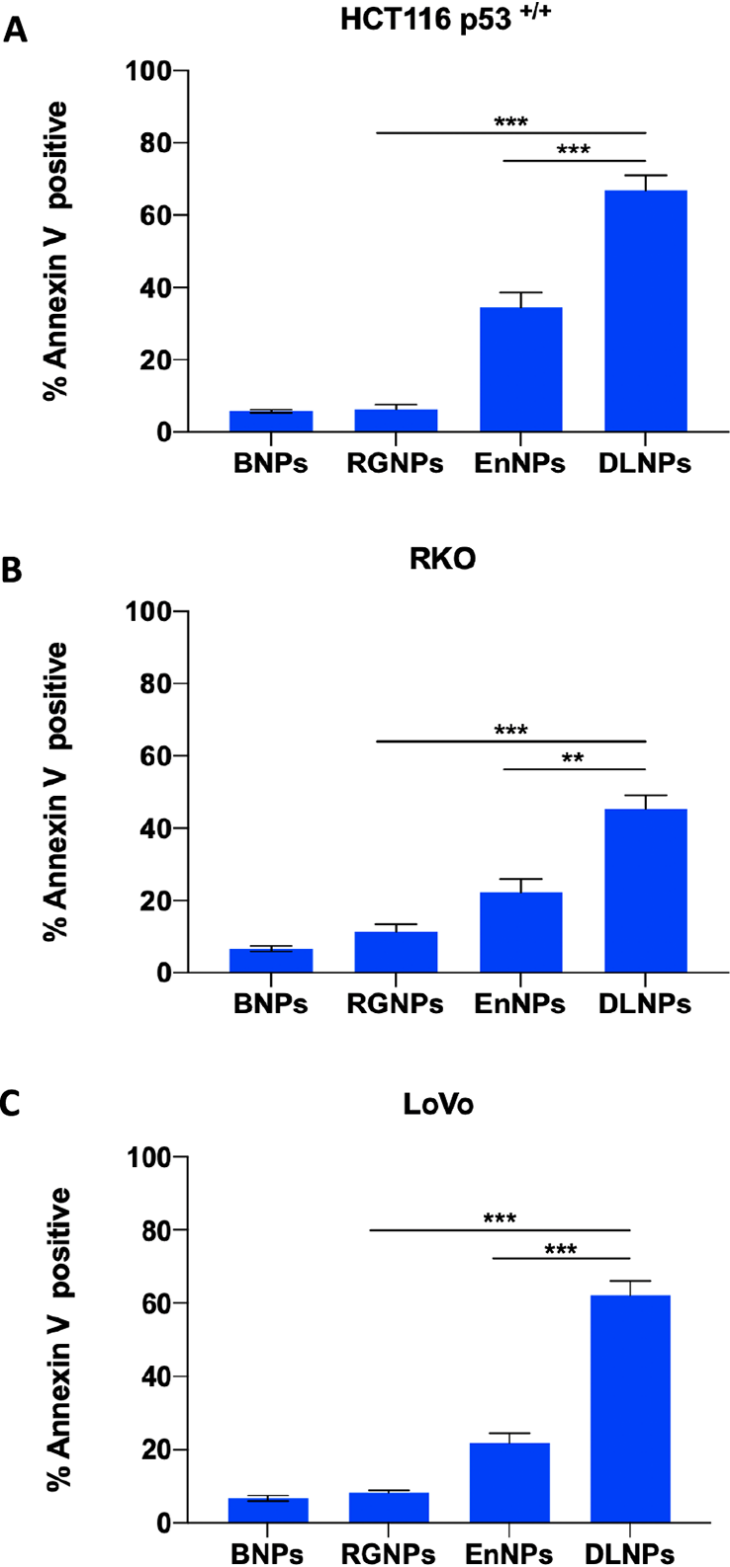
DLNPs induce apoptosis in colorectal cancer cells. HCT116 p53^+/+^ (A), RKO (B), and LoVo (C) cell lines were treated with RGNPs (equating to 1 μM of RG7388), EnNPs (equating to 2 μM of Entinostat), DLNPs (equating to 1 μM of RG7388 and 2 μM of Entinostat), or BNPs (equating to polymer concentration of DLNPs) for 72 h prior to Annexin-V/PI flow cytometry analysis. **p < 0.01, ***p < 0.001 calculated by one-way ANOVA (Tukey posthoc). Data expressed as mean ± SD of three independent experiments.

**Figure 6:**
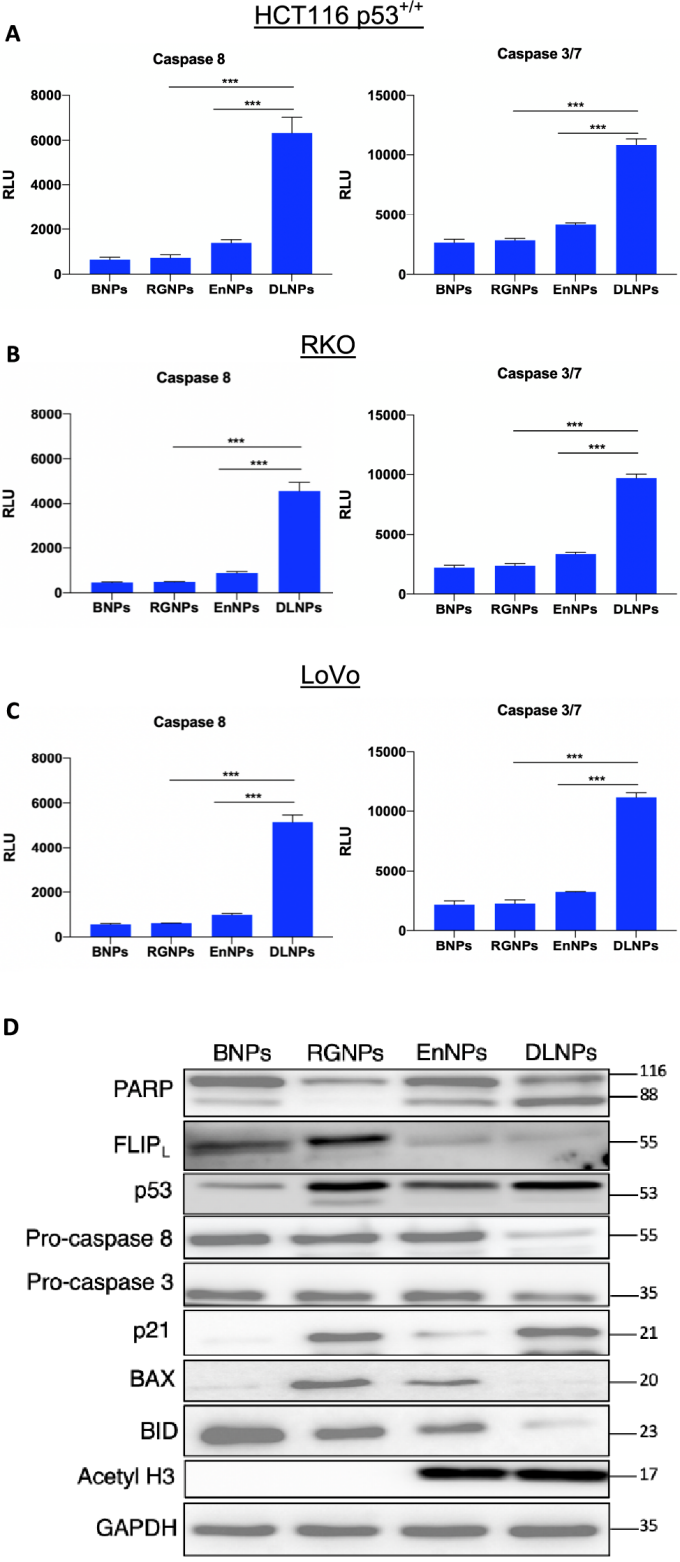
DLNPs induce apoptosis through FLIP_L_ downregulation. HCT116 p53^+/+^ (A), RKO (B), and LoVo (C) cell lines were treated with RGNPs (equating to 1 μM of RG7388), EnNPs (equating to 2 μM of Entinostat), DLNPs (equating to 1 μM of RG7388 and 2 μM of Entinostat), or BNPs (equating to polymer concentration of DLNPs) for 72 h. Lysates were then collected, and caspase activity assays were performed. ***p < 0.001 calculated by one-way ANOVA (Tukey posthoc). Western blot analysis for the indicated proteins was performed following treatment of HCT116 p53^+/+^ cells with the same drug regimen (D). Data expressed as mean ± SD of three independent experiments.

Following successful coencapsulation of RG7388 and Entinostat within a single NP formulation, we next examined the effects on colorectal cell lines treated with RGNPs, EnNPs, or DLNPs (ensuring that RG7388 and Entinostat concentrations were equivalent across single and dual formulations) by analysis of cell death with Annexin-V/PI flow cytometry (Figure 5). Minimal apoptosis induction was observed in RGNP- and EnNP-treated cell lines, whereas treatment with the DLNPs resulted in significantly greater levels of apoptosis. Significantly higher levels of caspase 8 and caspase 3/7 activity were observed in DLNP-treated cells compared to the single agent nanoformulations supporting induction of apoptosis (Figure 6 A–C). Western blot analysis on lysates extracted from HCT116 p53^+/+^ cells (Figure 6 D) revealed a marked upregulation in p53 and its canonical target p21 in response to treatment with RGNPs and DLNPs, indicating the activity of encapsulated RG7388. Similarly, acetyl H3 upregulation was observed upon EnNPs and DLNPs exposure, indicating the activity of Entinostat. Importantly, aligned with our recent publication,^9^ potent suppression of FLIP(L) was observed in EnNP- and DLNP-treated cells, which coincided with increases in activation of caspases 3/8, as well as BID and PARP cleavage, indicative of enhanced cell death.

### Coencapsulation of RG7388 and Entinostat Limits Hematological Toxicity

**Figure 7:**
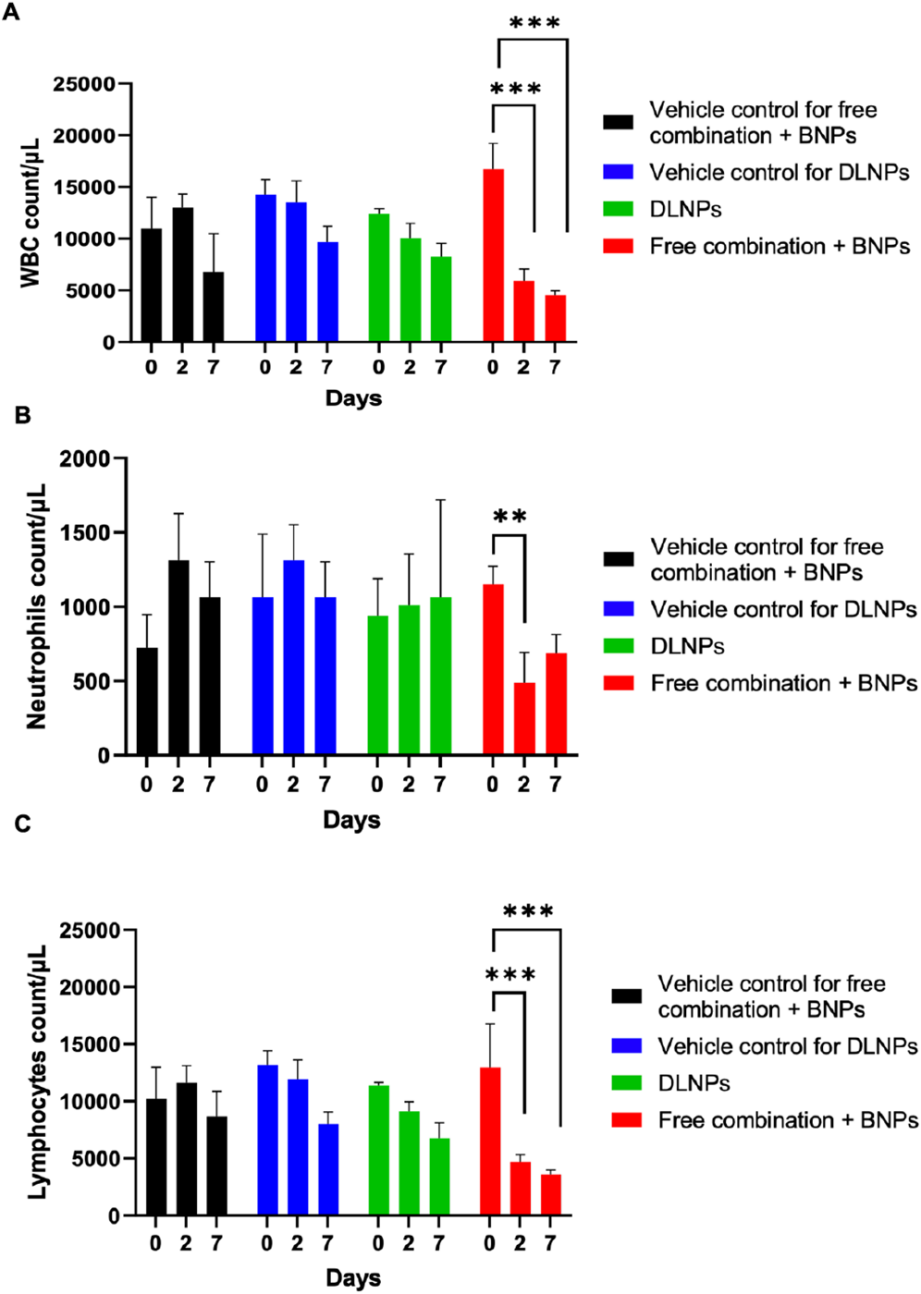
Nanoencapsulation of RG7388 and Entinostat within DLNPs results in reduced systemic toxicity against WBCs. C57BL/6 mice were treated with two doses (at days 0 and 5) of DLNPs via intravenous injection, equivalent doses of free drugs mixed with BNPs via intraperitoneal injection, or corresponding vehicle controls. Blood samples were taken at baseline (day 0) and 48 h after each dose (days 2 and 7), and total WBCs (A), neutrophils (B), and lymphocytes (C) were quantified. **p < 0.01, ***p < 0.001 calculated by two-way ANOVA (Tukey posthoc). Data expressed as mean ± SEM.

Hematological toxicities such as thrombocytopenia and leukopenia represent the most common and serious adverse effects associated with RG7388 and Entinostat treatment. To test if nanoencapsulation of both agents would result in a reduction in these toxicities, we compared the effects of treating C57BL/6 mice with DLNPs with equivalent doses of free RG7388, Entinostat, and blank nanoparticles (BNPs) or vehicle controls. Analysis of various hematological parameters in blood samples collected at 48 h following treatment indicated that the free RG7388/Entinostat/BNP combination resulted in a significant reduction in WBC count compared to the baseline (Figure 7A). In contrast, no significant reduction in the WBC count was observed in response to treatment with DLNPs, highlighting the protective effects of this nanoparticle-based treatment approach. To obtain further insights into the effects of the drug combination on subpopulations of WBCs, differential blood counts were then performed. Treatment with the free drug combination led to significant reductions in both neutrophil (Figure 7B) and lymphocyte (Figure 7C) numbers compared to baseline levels. Again, however, these effects were largely curtailed following the nanoencapsulation of both agents, with no significant reductions in neutrophils and lymphocytes following DLNPs therapy. Animal body weight was monitored throughout the study as an indicator of treatment tolerability (Supplementary Figure 9). All weights remained consistent throughout the study, suggesting that the treatments were well tolerated.

### Discussion

In the current study, we have demonstrated the capability to generate PLGA nanoparticles that coencapsulate the MDM2 antagonist RG7388 and the Class-I HDAC inhibitor Entinostat at a controlled ratio that elicits synergistic cell death in a panel of colorectal cancer cell models.

MDM2 antagonists such as RG7388 are regarded as promising agents to treat cancer. However, their application is hampered by several issues, such as dose-limiting toxicities and poor pharmacokinetic profiles.^18,19^ Reformulation of the drug represents a possible approach to reduce these effects, and indeed other compounds such as nutlin-3a have been evaluated for potential in nanoformulations.^20^ Herein, we showed that we could successfully encapsulate RG7388 within PEGylated PLGA NPs. Biological evaluation of these nanoparticles demonstrated that the formulation yielded comparable therapeutic effects to the nonencapsulated drug and was able to induce cell cycle arrest. However, the formulation was unable to induce apoptosis in our models. The cell fate decision (apoptosis versus cell cycle arrest) in response to MDM2 antagonists remains unclear. However, the primary response to p53 activation in most hematologic tumors with wild-type p53 is apoptosis, whereas most solid tumors only undergo cell cycle arrest,^17,21−24^ which is consistent with our findings.

Recently, we have found that the antiapoptotic protein FLIP_L_ is upregulated in colorectal cancers and is a critical mediator of resistance to RG7388.^9^ This previous work showed that Entinostat could reduce the expression of FLIP_L_ and enhance the potency of RG7388. We, therefore, undertook a detailed analysis of the potential additive effects of the two agents and established synergistic ratios of the two agents, finding an optimal RG7388:Entinostat molar ratio of 1:2.

Despite having determined the optimal synergistic ratio between RG7388 and Entinostat *in vitro*, this may not necessarily translate therapeutically due to the dissimilar pharmacokinetic profiles of each drug. Moreover, previous studies and clinical trials have revealed dose-limiting systemic toxicities associated with the administration of HDAC inhibitors and MDM2 antagonists, specifically hematological toxicities such as thrombocytopenia and leukopenia.^25,26^ These side effects are not surprising given the crucial role of p53 and HDACs in hematopoiesis regulation.^27−29^ Thus, we next sought to coentrap both agents within a single nanoformulation with the aim of unifying their pharmacokinetics and mitigating toxicity. While it may also be possible to employ a mixture of separately encapsulated drugs, this may not necessarily ensure concurrent delivery of both agents to tumors at their synergistic ratio.^30,31^ We demonstrated that RG7388 and Entinostat could be simultaneously loaded within PLGA NPs at the optimal molar ratio that was previously determined. Assessment of drug release from the NPs showed that both agents were released in a similar pattern, and that the optimal synergistic ratio was maintained throughout the study. Moreover, the DLNPs were effective against colorectal cancer models, as indicated by their ability to elicit synergistic cell death. Importantly, we also confirmed that *in vivo* hematological toxicity was markedly reduced upon administration of entrapped versus free agents, exemplifying the protective effects of nanoformulation. This may be explained by the controlled release kinetics of RG7388 and Entinostat from the nanoparticles, in contrast to the sharp rise in drug plasma concentrations that would likely be seen with RG7388 and Entinostat in a free format. These findings mirror previous work from our laboratory examining the coencapsulation of ABT-737 and camptothecin within PEG–PLGA NPs.^32^ Moreover, the nanoencapsulation of these agents significantly reduced the occurrence of systemic side effects such as leukopenia, thrombocytopenia, and GI adverse effects.^32^ Tian et al. also investigated the coencapsulation of paclitaxel and cisplatin within polymeric NPs. This coencapsulation resulted in more pronounced tumor growth inhibition compared to the free drug combination against nonsmall cell lung cancer cells *in vivo*.^33^ Collectively, these developments clearly highlight the ability of nanotechnology to improve therapeutic outcomes and overcome many hurdles associated with combination cancer therapy, such as inadequate tumor deposition of synergetic drug amounts and dose-limiting toxicities.^34^ Indeed, the success of this approach has now been exemplified through the FDA approval of the Vyxeos nanoformulation (1:5 of daunorubicin and cytarabine) in August 2017 for the treatment of AML.^35^ CPX-1, which is another liposomal-based formulation coencapsulating irinotecan and floxuridine (1:1), is also now in phase II clinical trials for the treatment of advanced colorectal cancer.^36^

In summary, while RG7388 exhibits potent activity in inducing cell cycle arrest, its ability to induce cell death as a stand-alone agent remains modest. However, we demonstrate its potential efficacy in anticancer therapy when combined with Entinostat in a combination regimen. Our work provides the first demonstration of the successful coencapsulation of RG7388 and Entinostat within polymeric NPs at their optimal synergistic ratio. We show that drug efficacy is maintained following nanoformulation, leading to synergistic induction of cell death in a panel of colorectal models *in vitro*. Moreover, we also demonstrate that *in vivo* administration of RG7388 and Entinostat in a free format leads to leukopenia, which can be largely mitigated upon nanoformulation of both agents. These findings clearly highlight the benefits that nanotechnology can offer toward combined chemotherapy administration and warrant future PK and animal efficacy studies to evaluate the DLNPs further.

## Supporting Information Available

Supplementary Figure 1: development of an analytical method to quantify Entinostat content within DLNPs, Supplementary Figure 2: characterization of RGNPs and EnNPs, Supplementary Figure 3: assessment of NPs uptake in colorectal cancer cells by confocal microscopy and flow cytometry, Supplementary Figure 4: representative histograms depicting the effect of RGNPs on cell cycle distribution in colorectal cancer cell lines, Supplementary Figure 5: dose response curves for free RG7388 and RGNPs in colorectal cancer cell lines, Supplementary Figure 6: dose response curves for free RG7388 and free Entinostat alone or in combination in colorectal cancer cell lines, Supplementary Figure 7: Annexin-V/PI flow cytometry analysis of EnNPs, Supplementary Figure 8: SEM analysis of DLNPs, Supplementary Figure 9: body weight assessment following cotreatment with RG7388 and Entinostat either as free drugs or within DLNPs (PDF)
