# Supplementary material for: Nanoencapsulation of MDM2 Inhibitor RG7388 and Class-I HDAC Inhibitor Entinostat Enhances their Therapeutic Potential Through Synergistic Antitumor Effects and Reduction of Systemic Toxicity
Source: Mol Pharm. 2024 Feb 9;21(3):1246–55. doi: 10.1021/acs.molpharmaceut.3c00926 (PMC10915795; doi:10.1021/acs.molpharmaceut.3c00926)
Supplement: Supplementary file 1 — mp3c00926_si_001.pdf [file mp3c00926_si_001.pdf]

Title

Nanoencapsulation of MDM2 inhibitor RG7388 and Class-I HDAC inhibitor Entinostat enhances their therapeutic potential through synergistic antitumor effects and reduction of systemic toxicity

Authors

Anas Abed[1,2], Michelle K. Greene[1], Alhareth A. Alsa'd[2,3], Andrea Lees[1], Andrew Hindley[4], Daniel B Longley[1], Simon S McDade[1], Christopher J. Scott[1*]

Affiliations

[1] The Patrick G Johnston Centre for Cancer Research, School of Medicine, Dentistry and Biomedical Sciences, Queen's University Belfast, 97 Lisburn Road, Belfast, BT9 7AE, United Kingdom

[2] Pharmacological and Diagnostic Research Centre, Faculty of Pharmacy, Al-Ahliyya Amman University, Amman, 19111, Jordan

[3] School of Pharmacy, Queen's University Belfast, 97 Lisburn Road, Belfast, BT9 7BL, United Kingdom

[4] Clinical Haematology, Belfast City Hospital, 97 Lisburn Road, Belfast, BT9 7AB, United Kingdom

***Corresponding author**

c.scott@qub.ac.uk

Supplementary figures

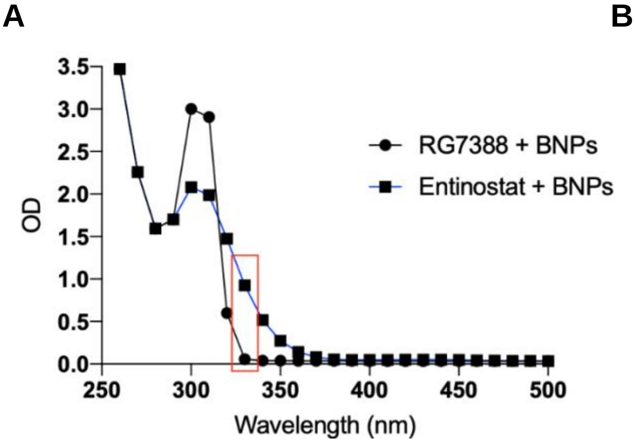

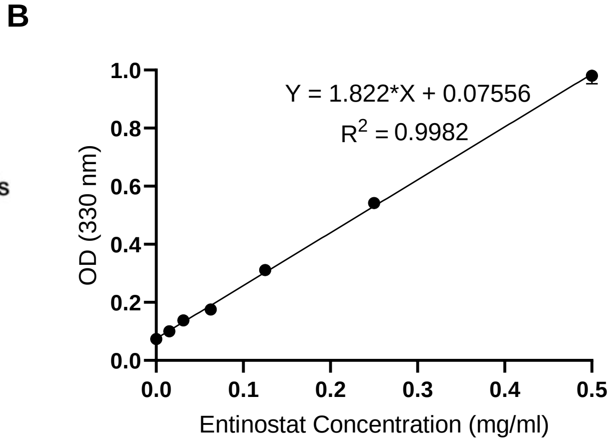

Supplementary Figure 1. Development of an analytical method to quantify Entinostat content within DLNPs. (**A**) Free Entinostat or free RG7388 were mixed with BNPs in 1 mL of 1:1 ACN:DMSO prior to UV-VIS spectrum analysis. A wavelength of 330 nm (indicated by red box, where RG7388 interference was negligible) was selected for subsequent development of an Entinostat quantification method in (**B**). Entinostat standard curve was then prepared by spiking known amounts of free Entinostat into BNPs in 1:1 ACN:DMSO with the highest concentration corresponding to 100% entrapment efficiency. OD = Optical Density.

A

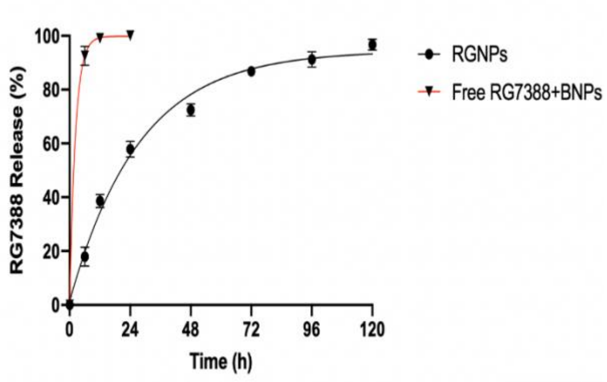

B

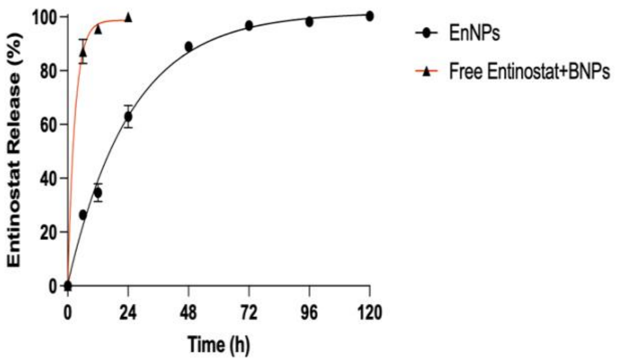

Supplementary Figure 2. Characterisation of RGNPs and EnNPs. Drug release profile of RGNPs (**A**) and EnNPs (**B**). 20 mg of RGNPs or EnNPs were resuspended in 1 mL of PBS and then injected into a dialysis cassette which was then immersed in PBS containing 10% FBS and 1% Tween-20 under constant stirring at 37°C. At the indicated timepoints, the remaining NPs were removed and drug release was quantified. Data represents mean ± SEM of three independent experiments.

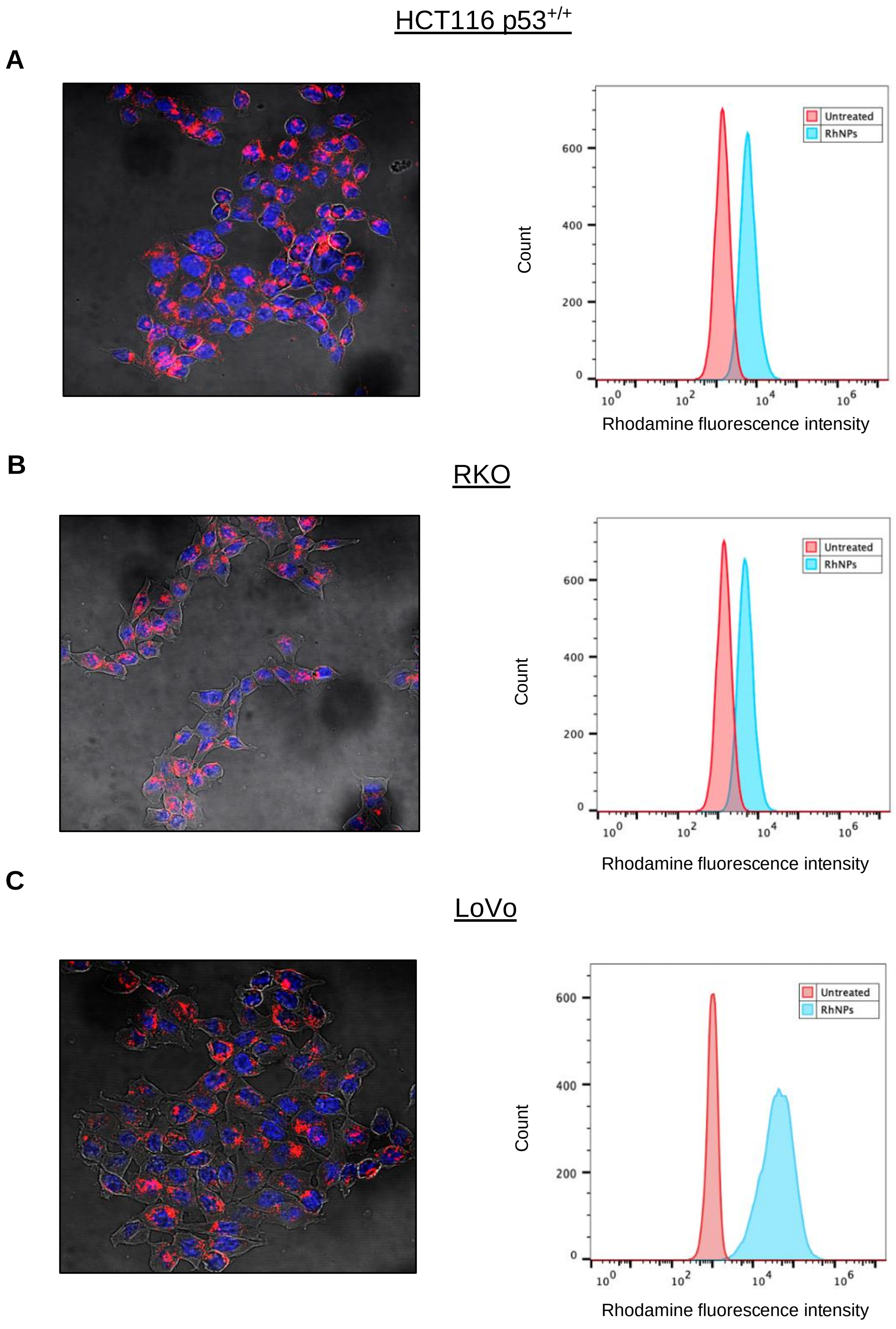

Supplementary Figure 3. Assessment of NPs uptake in colorectal cancer cells by confocal microscopy and flow cytometry. HCT116 p53$^{+/+}$ (**A**), RKO (**B**) and LoVo (**C**) cells were treated with 200 µg/mL of Rhodamine B-loaded NPs for 6 hours prior to flow cytometry and confocal microscopy analyses.

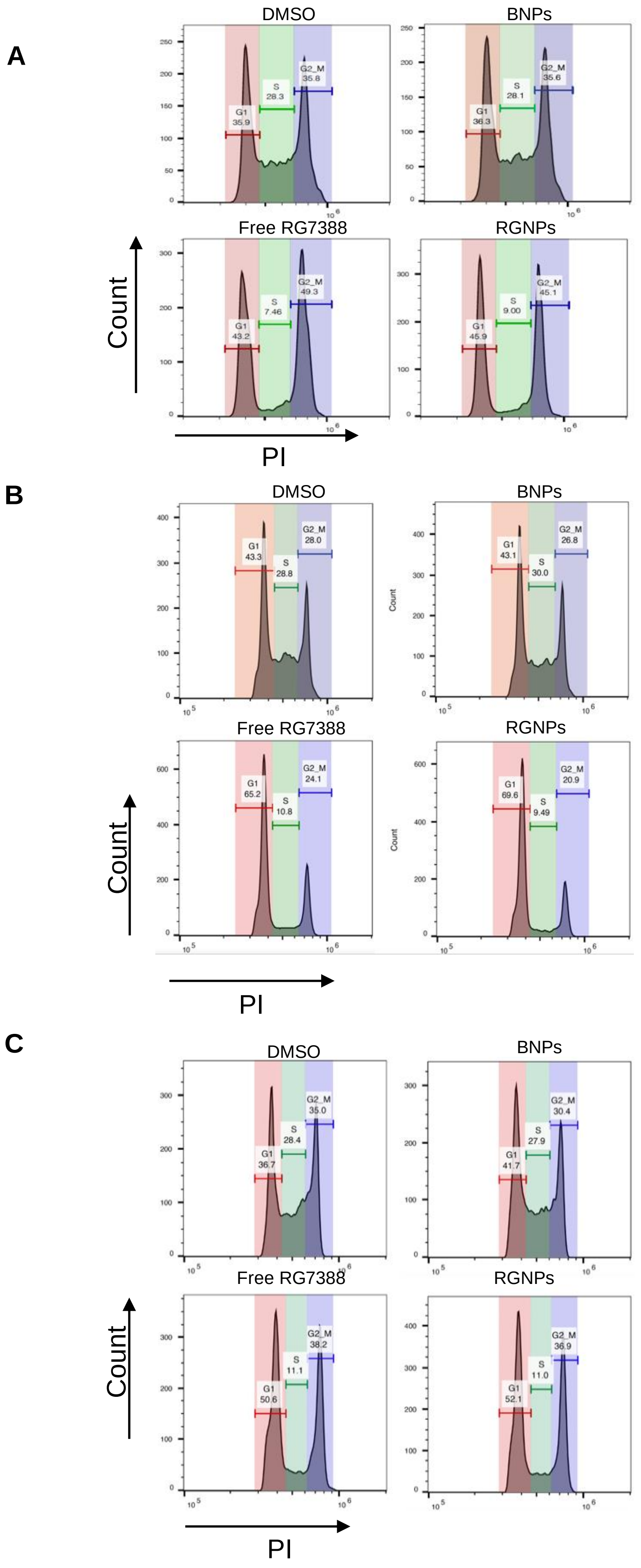

Supplementary Figure 4. Representative histograms depicting the effect of RGNPs on cell cycle distribution in colorectal cancer cell lines. HCT116 p53$^{+/+}$ (**A**), RKO (**B**) and LoVo (**C**) cell lines were treated with 1 µM of free RG7388, RGNPs (equating to 1 µM of RG7388) or BNPs (equating to polymer concentration of RGNPs) for 24 hours prior to cell cycle analysis by flow cytometry. Data expressed as mean ± SD of three independent experiments.

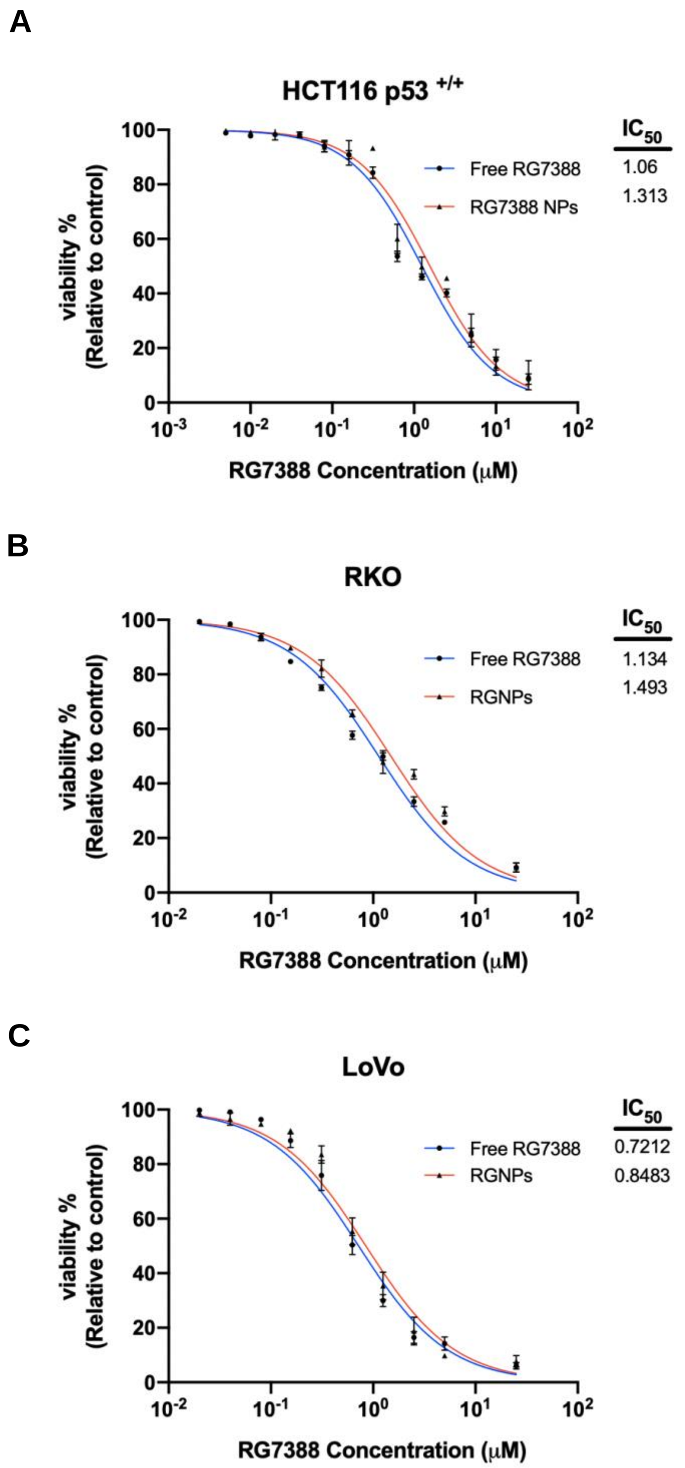

Supplementary Figure 5. Dose response curves for free RG7388 and RGNPs in colorectal cancer cell lines. HCT116 $p53^{+/+}$ **(A)**, RKO **(B)** and LoVo **(C)** cell lines were treated with serial dilutions of free RG7388 or RGNPs for 72 hours prior to analysis using the CellTiter-Glo® viability assay. Graphs are presented as percentage viability relative to the untreated control. Data represents mean ± SEM of three independent experiments.

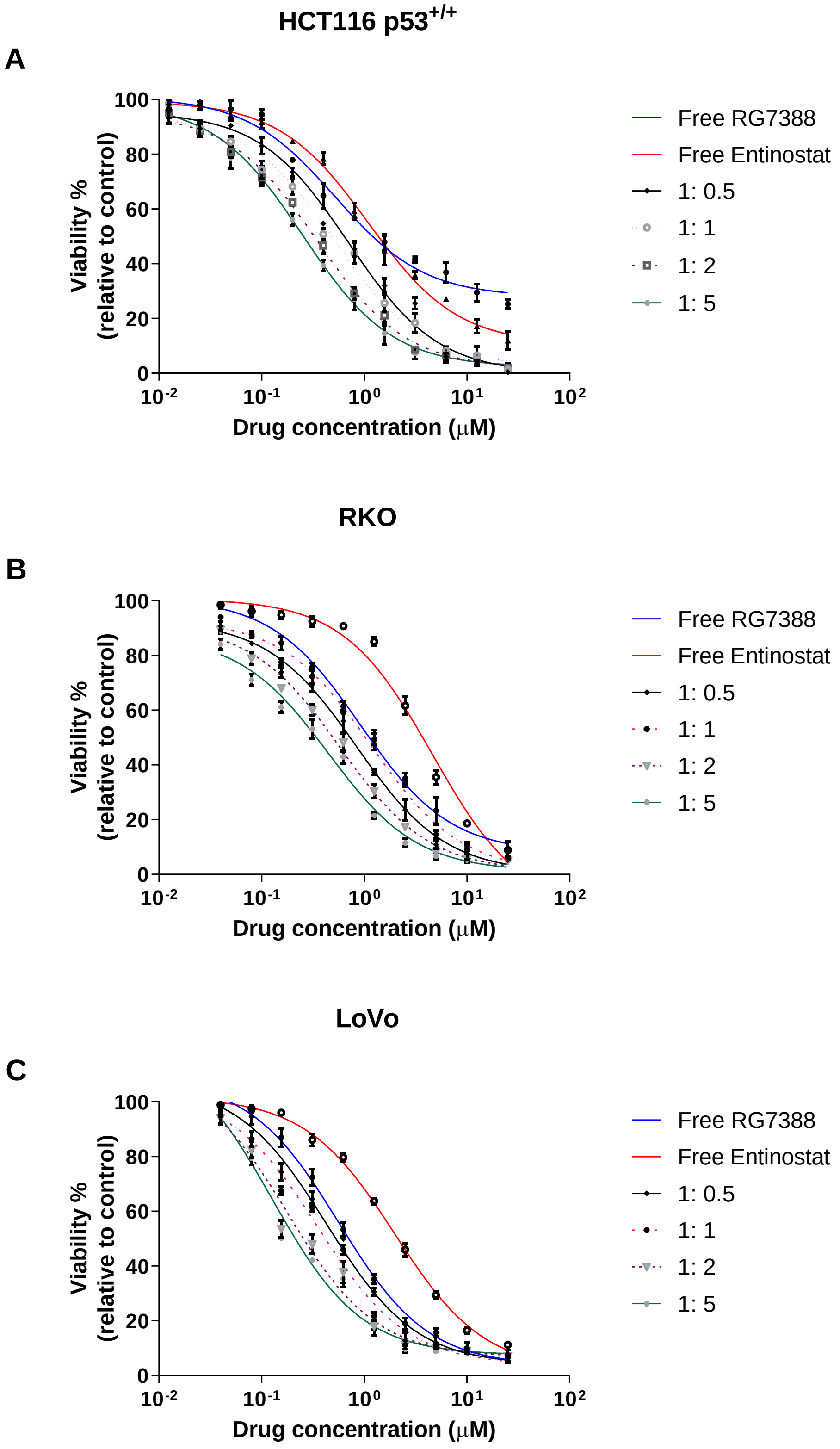

Supplementary Figure 6. Dose response curves for free RG7388 and free Entinostat alone or in combination in colorectal cancer cell lines. HCT116 $p53^{+/+}$ **(A)**, RKO **(B)** and LoVo **(C)** cell lines were treated with a concentration range of free RG7388 and free Entinostat as monotherapy or in combination at various molar ratios of RG7388:Entinostat for 72 hours prior to analysis using the CellTiter-Glo® viability assay. Graphs are presented as percentage viability relative to the untreated control. Figure keys denote RG7388:Entinostat molar ratios. Data represents mean ± SEM of three independent experiments.

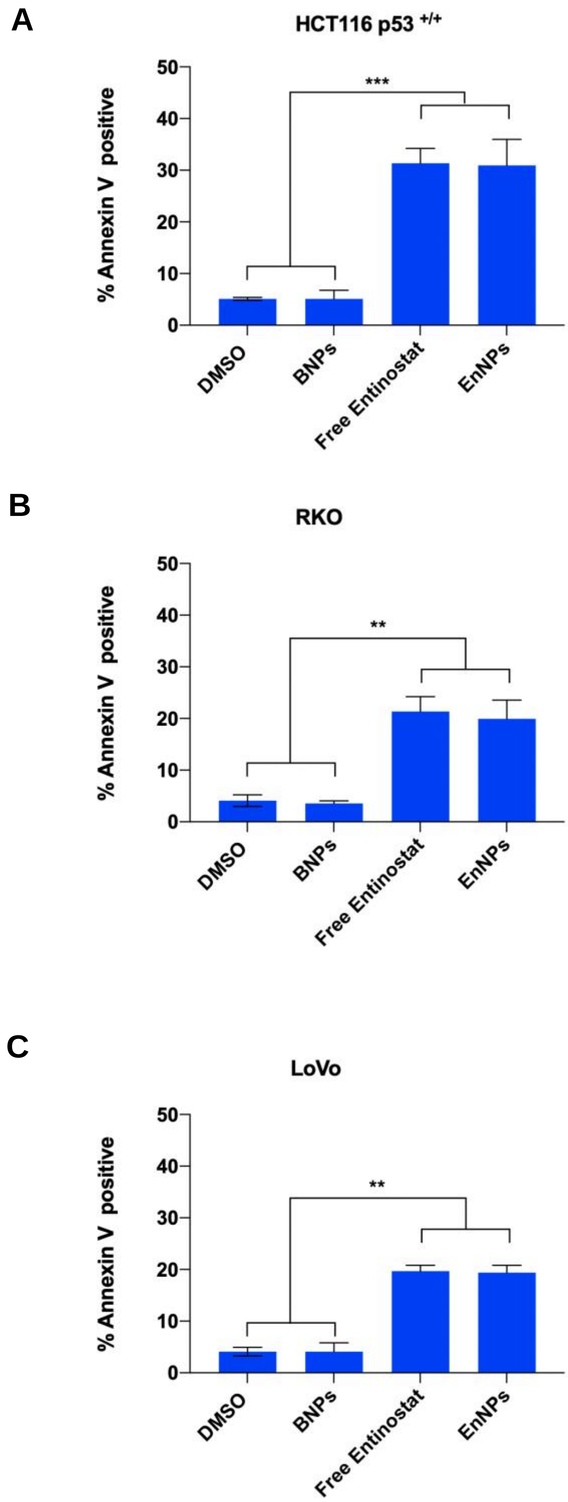

Supplementary Figure 7. Annexin-V/PI flow cytometry analysis of EnNPs. HCT116 p53$^{+/+}$ **(A)**, RKO **(B)** and LoVo **(C)** cell lines were treated with 2.5 µM of free Entinostat, EnNPs (equating to 2.5 µM of Entinostat) or BNPs (equating to polymer concentration of EnNPs) for 72 hours prior to Annexin-V/PI flow cytometry analysis. **$p < 0.01$, ***$p < 0.001$ calculated by one-way ANOVA (Tukey post-hoc). Data represents mean ± SD of three independent experiments.

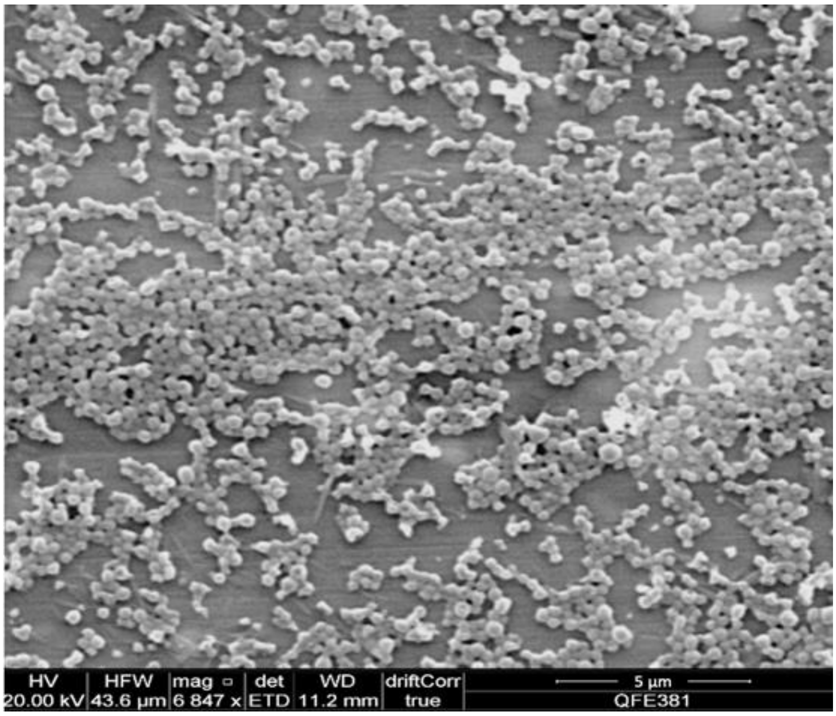

Supplementary Figure 8. SEM analysis of DLNPs. Scale bar = 5 µm.

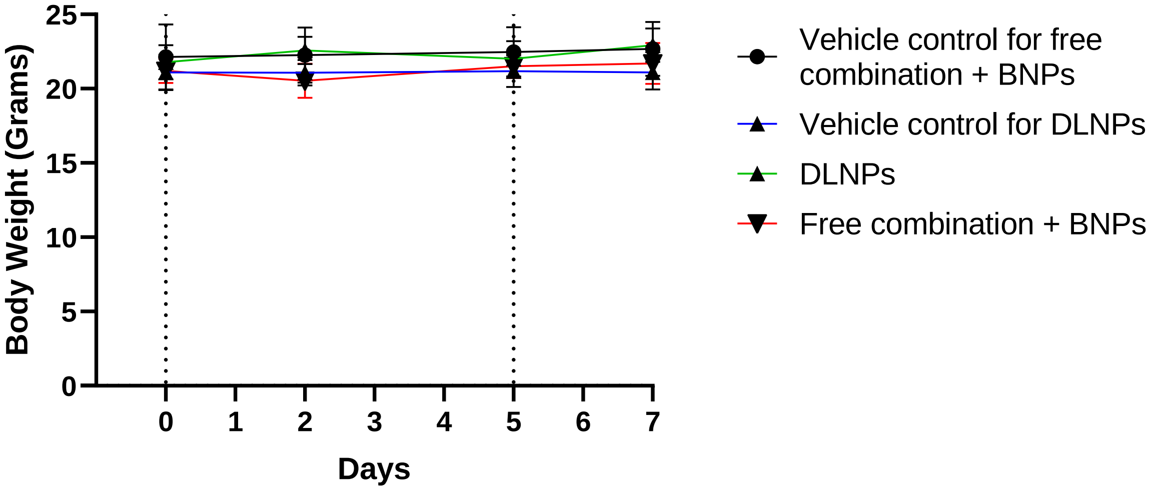

Supplementary Figure 9. Body weight assessment following co-treatment with RG7388 and Entinostat either as free drugs or within DLNPs. C57BL/6 mice were treated with two doses (at days 0 and 5) of DLNPs via intravenous injection, equivalent doses of free drugs mixed with BNPs via intraperitoneal injection or corresponding vehicle controls. Body weights were routinely monitored throughout the study. Data represents mean ± SEM.
